# Phenotyping misophonia: Psychiatric disorders and medical health correlates

**DOI:** 10.3389/fpsyg.2022.941898

**Published:** 2022-10-06

**Authors:** M. Zachary Rosenthal, Kibby McMahon, Anna S. Greenleaf, Clair Cassiello-Robbins, Rachel Guetta, Jacqueline Trumbull, Deepika Anand, Emily S. Frazer-Abel, Lisalynn Kelley

**Affiliations:** ^1^Department of Psychiatry and Behavior, Duke University Medical Center, Durham, NC, United States; ^2^Department of Psychology and Neuroscience, Duke University, Durham, NC, United States; ^3^Triangle Area Psychology Clinic, Durham, NC, United States; ^4^Chicago Center for CBT, Chicago, IL, United States

**Keywords:** misophonia, mental health, medical history, psychiatric disorders, anxiety disorders

## Abstract

Misophonia is characterized by decreased tolerance to specific sounds and associated stimuli that causes significant psychological distress and impairment in daily functioning (Swedo et al., 2022). Aversive stimuli (often called “triggers”) are commonly repetitive facial (e.g., nose whistling, sniffling, and throat clearing) or oral (e.g., eating, drinking, and mouth breathing) sounds produced by other humans. Few empirical studies examining the nature and features of misophonia have used clinician-rated structured diagnostic interviews, and none have examined the relationship between misophonia and psychiatric disorders in the Diagnostic and Statistical Manual-5th version (DSM-5; American Psychiatric Association, 2013). In addition, little is known about whether there are any medical health problems associated with misophonia. Accordingly, the purpose of the present study was to improve the phenotypic characterization of misophonia by investigating the psychiatric and medical health correlates of this newly defined disorder. Structured diagnostic interviews were used to assess rates of lifetime and current DSM-5 psychiatric disorders in a community sample of 207 adults. The three most commonly diagnosed current psychiatric disorders were: (1) social anxiety disorder, (2) generalized anxiety disorder, and (3) specific phobia. The three most common lifetime psychiatric disorders were major depressive disorder, social anxiety disorder, and generalized anxiety disorder. A series of multiple regression analyses indicated that, among psychiatric disorders that were correlated with misophonia, those that remained significant predictors of misophonia severity after controlling for age and sex were borderline personality disorder, obsessive compulsive disorder, and panic disorder. No medical health problems were significantly positively correlated with misophonia severity.

## Introduction

Misophonia is a recently defined disorder characterized by decreased tolerance to specific sounds and associated stimuli that causes significant psychological distress and impairment in daily functioning (Swedo et al., 2022). Generally, these aversive stimuli (often called “triggers”) are commonly repetitive facial (e.g., nose whistling, sniffling, and throat clearing) or oral (e.g., eating, drinking, and mouth breathing) sounds produced by other humans. Ideographically, however, there are individual level differences in the types of cues (e.g., repetitive visual stimuli, objects, and environmental sounds) and contexts (e.g., the same stimulus may have different effects when produced by specific people) associated with misophonia (for reviews, see Brout et al., 2018; Potgieter et al., 2019).

When an individual with misophonia anticipates or directly encounters triggering stimuli, common responses include physiological arousal (e.g., sympathetic nervous system activation), negatively valenced affective experiences (e.g., irritation, anger, anxiety, and disgust), and behavioral patterns congruent with freeze (e.g., hypervigilance toward possible trigger sources), flight (e.g., escape or avoidance behavior), and fight behaviors (e.g., indirect interpersonal aggression), all of which may be experienced as highly distressing and distinct from what would be expected typically by others in such contexts (for a recent comprehensive review, see Swedo et al., 2022). This multi-modal breadth of responses is, notably, incongruent with the literal translation of the term misophonia (i.e., hatred or dislike of sound). Put differently, in spite of its denotation, misophonia symptoms are neither limited to the emotion of hate (or other anger-related affective states) nor solely elicited by sounds.

Although scientific research investigating misophonia began less than 10 years ago (Edelstein et al., 2013; Schröder et al., 2013), the term has been in use for over 20 years. Other synonymous terms were used (e.g., selective sound sensitivity) prior to misophonia being coined by Jastreboff and Jastreboff (2001) and subsequently adopted by the lay public, clinicians, and scientists. More generally, misophonia has been framed within a broader category of disorders characterized by decreased sound tolerance (Jastreboff and Jastreboff, 2014). Similar but distinct conditions include hyperacusis (i.e., the physical properties of sounds, rather than their contextually associated meaning, are experienced as excessively intense and distressing), tinnitus (i.e., aversive ringing in the ears), and phonophobia (i.e., fear and avoidance of certain sounds). Additionally, some have used the term “annoyance hyperacusis” in a manner that is highly similar to misophonia (Tyler et al., 2014). Despite conceptual and definitional overlap of language used historically, the recently published consensus definition (Swedo et al., 2022) lays the groundwork for the term misophonia to be used, moving forward, in a more clear and consistent manner across a wide range of stakeholders (e.g., community members, clinicians, and researchers).

Recent empirical research has begun to identify phenotypic features associated with misophonia, which may help inform understanding of the etiology and maintenance of the condition and is important for developing treatment strategies. Although several studies have used neuroimaging or other experimental methods to identify candidate neural mechanisms (e.g., Kumar et al., 2017, 2021; Eijsker et al., 2021), the vast majority of findings to date have come from self-report measures and clinician-rated interviews used to explore the phenotypic correlates of misophonia. No epidemiologic or longitudinal research has been conducted and very few studies have used clinical control conditions to differentiate misophonia from other conditions (for an exception, see Siepsiak et al., 2022). There is a significant need for studies with children, adolescents, and families to begin understanding the developmental vulnerabilities and etiological pathways by which misophonia begins.

To date, most of the published research examining the nature and features of misophonia has explored the relationship between misophonia and problems with mental health. Collectively, literature reviews on these topics suggest that misophonia symptoms are associated with greater psychopathology across a wide range of psychiatric disorders (Brout et al., 2018; Potgieter et al., 2019; Swedo et al., 2022). Examples of specific studies using self-report methodologies have found that misophonia symptom severity is positively correlated with neuroticism, anxiety symptoms, depressive symptoms, difficulties with emotion regulation, affective instability, anxiety sensitivity, certain obsessive compulsive disorder (OCD) symptoms, perfectionism, somatic pain, and a self-reported diagnosis of post-traumatic stress disorder (PTSD; Wu et al., 2014; McKay et al., 2017; Cusack et al., 2018; Quek et al., 2018; Rouw and Erfanian, 2018; Jager et al., 2020; Siepsiak et al., 2020b; Cassiello-Robbins et al., 2021; Guetta et al., 2022a). Additionally, using a household sampling approach in Turkey, a recent study found that adults with misophonia were significantly more likely than those without misophonia to self-report a lifetime history of attention deficit/hyperactivity disorder (ADHD), OCD, bipolar disorder, substance use disorder, and conversion disorder (Kılıç et al., 2021). When considered collectively, these studies point to the early conclusion that misophonia may be associated with various psychological processes and symptoms across a range of mental health problems, and not to any one specific disorder or category of disorders.

However, as may be expected for a new and understudied disorder with little support from extramural funding entities, the vast majority of these studies have been limited by a range of methodological problems which preclude clear or definitive inferences. Examples include limitations with sampling (e.g., small samples, convenience samples, online sampling limited to those using misophonia support groups), scope (e.g., assessment restricted to a subset of mental health problems), and measurement. A major limitation related to measurement in many studies of misophonia has been the reliance on self-report inventories (i.e., surveys) with little to no psychometrically reported reliability or validity. Nonetheless, recently published research in the last 2 years has yielded several new self-report measures of misophonia with strong initial psychometric support. Examples include the Amsterdam Misophonia Scale (AMISOS-R; Schröder et al., 2013), Duke Misophonia Questionnaire (DMQ; Rosenthal et al., 2021), MisoQuest (Siepsiak et al., 2020a), Misophonia Response Scale (MRS; Dibb et al., 2021), and S-Five (S5; Vitoratou et al., 2021).

Each of these self-report measures has shown preliminary psychometric validation, and, collectively, provides promising new tools to help clinicians and researchers characterize misophonia. Despite the careful attention to psychometric considerations in the development of these measurement tools, additional studies are needed to cross-validate findings and provide clearer support for the sensitivity and specificity of these measures in the assessment of misophonia specifically, and not to other related phenomena. In addition, only one published study has demonstrated preliminary psychometric support for a structured clinical interview assessing misophonia (Duke Misophonia Interview; Guetta et al., 2022a), and no self-report or interview measures have been *a priori* developed and validated using the recently published consensus definition of misophonia (Swedo et al., 2022).

Although self-report assessment measures may be easy to access and administer, can be brief and quickly scored, there are many problems with relying on subjective measurement approaches alone when examining candidate phenotypic features of a newly defined construct. Weaknesses of self-report measures include poorly worded items (e.g., compound questions, items with jargon), items that do not fully measure the construct of interest, varying interpretations of items, response biases, limitations in knowledge or insight about items, and demand characteristics associated with the measure. Such problems with self-report are not unique to research on misophonia. Furthermore, however nascent that research on misophonia may be, reliance on self-report measurement has yielded information that is informative in generating hypotheses about the nature of misophonia. Following self-report measurement, a next step in advancing an understanding of the mental health problems associated with misophonia is the use of clinician-rated structured diagnostic interviews.

Several recent studies have used such measurement approaches. Jager et al. (2020) conducted the largest and most rigorous study to date examining the relationship between misophonia and psychiatric disorders in adults. In this study, 575 adults presenting for treatment at a clinic for misophonia in Amsterdam were interviewed using the Mini International Neuropsychiatric Interview (M.I.N.I.; Sheehan et al., 1998), a structured diagnostic assessment measure that assesses the presence of 15 current psychiatric disorders. Results from this study indicated that 72% of the sample did not meet the full criteria for any current psychiatric disorder. The most common current psychiatric disorders were mood disorders (10.1%), anxiety disorders (9%), ADHD (5.4%), and personality disorders (5%). Examples of other disorders that were less commonly observed were autism spectrum disorder (2.4%), substance use disorder (1.6%), impulse control disorder (2.1%), and tic disorder (1.6%). The findings from Jager et al. (2020) suggest that (a) a minority of adults seeking treatment for misophonia meet full criteria for any psychiatric disorder and (b) no singular disorder appears to be specifically related to misophonia.

In addition to mental health diagnoses, Jager et al. (2020) collected information about past medical history. Most participants (80%) reported no history of any medical health problems, and a small minority indicated having more than one medical health problem. Of those with medical health problems, the most common diagnoses were migraines, irritable bowel syndrome, asthma, and back pain. Additionally, hyperacusis (0.7%) and tinnitus (1.7%) were rarely reported medical conditions. Findings from Jager et al. (2020) about medical history suggest that misophonia may not be associated with any specific medical history problems. However, additional studies are needed before firm conclusions can be drawn.

The purpose of the present study was to replicate and extend the literature characterizing the nature and features of misophonia in adults. Specifically, the primary aim was to comprehensively investigate the relationship between misophonia severity and (a) categories of psychiatric disorders (e.g., mood, anxiety, etc.), (b) specific psychiatric disorders (e.g., major depressive disorder, PTSD, etc.), and (c) medical health history. This is the first large study to examine the associations between misophonia and DSM-5 psychiatric disorders using the SCID-5 (First et al., 2015a), a psychometrically validated, comprehensive structured psychiatric interview commonly used in large epidemiologic studies of psychiatric disorders. In addition, this is the first study we are aware of to report rates of lifetime medical health problems in adults with misophonia. Accordingly, results from this study may offer new insights into the mental health and medical history correlates of misophonia.

## Materials and methods

### Participants

Individuals between ages 18 and 65 enrolled in the study by accessing a link on the Duke Center for Misophonia and Emotion Regulation website^1^, which took them to an online screen conducted in REDCap (Harris et al., 2009). The study was approved by the Duke Health Institutional Review Board, and all participants provided signed informed consent to participate. No data are available indicating how participants learned about the study, but anecdotal reports suggest most individuals learned about the study from online sources (e.g., searching for information about misophonia, social media, and news media stories about misophonia linking to our Center). Participants received $75 for participation. Individuals who met the criteria for a current psychotic disorder, current mania, current anorexia, or were unable to read English were excluded from the online screen. There were 210 participants who completed eligibility screening and enrolled in the study. One person dropped out and two did not qualify to continue after meeting diagnostic criteria for current psychosis. Therefore, the final sample included 207 participants (females = 74.4%, *n* = 154) with an average age of 35.72 years (*SD* = 12.49). Detailed demographic information is provided in Table 1.

**TABLE 1:** Demographic characteristics.

Characteristic	Participant	
	
	*n*	%
Age *(M, SD)*	35.7	12.5
Sex		
Male	53	25.6
Female	154	74.4
Gender Identity		
Male	53	25.6
Female	150	72.5
Genderqueer	2	1.0
Other	1	0.5
Did not disclose	1	0.5
Sexuality		
Straight	166	80.2
Gay	8	3.9
Bisexual	17	8.2
Something else	8	3.9
Don’t know	7	3.4
Did not disclose	1	0.5
Race		
White	167	80.7
African American	9	4.3
Chinese	7	3.4
Other Asian	7	3.4
Middle Eastern	2	1.0
Other	5	2.4
More than one race	10	4.8
Hispanic/Latinx		
Yes	26	12.6
No	181	87.4
Country Born In		
United States	191	92.3
Europe	5	2.5
Latin America	3	1.5
China	3	1.4
South Asia	4	1.9
Other	1	0.5
Income Level		
0–$10,000	29	14.0
10,001–$65,000	69	33.3
65,001–more than $100,000	109	52.7
Marital Status		
Single	91	44.0
Widowed	3	1.4
Married	77	37.2
Separated	3	1.4
Divorced	9	4.3
Living with partner	23	11.1
Missing	1	0.5

*N* = 207.

### Measures

#### Structured clinical interview for diagnostic and statistical manual-5th, research version

The structured clinical interview for DSM-5 (SCID-5) is a psychometrically validated semi-structured interview and was used to assess current and lifetime symptoms of DSM-5 disorders by a trained assessor (First et al., 2015b). Variables used in this study included categorical diagnoses of DSM-5 current disorders of adulthood (e.g., in the past month or past 6 months) and history of these disorders across lifetime. Composite variables were also calculated to capture whether participants met the criteria for current categories of disorders, including obsessive-compulsive (OC Disorder; e.g., OCD, hoarding, etc.), mood (e.g., major depressive disorder, persistent depressive disorder, bipolar disorder, etc.), anxiety (e.g., generalized anxiety disorder, specific phobia, panic disorder, etc.), eating (e.g., anorexia, bulimia, etc.), substance use disorder (e.g., alcohol use disorder, etc.), or trauma-related disorder (e.g., PTSD). All diagnostic variables were coded dichotomously as 0 (below threshold and did not meet criterion) or 1 (above threshold and met the full criteria for the presence of disorder). Inter-rater reliability was assessed by a blind rater randomly rating 8% of SCID-I interviews *via* recorded interviews. Significant Cohen’s κ ranged from 0.63 to 1.00 (all *ps* < 0.05) for most disorders, reflecting acceptable inter-rater reliability. However, due potentially to the low rate of observed values in randomly selected interviews, Cohen’s κ was not significant for lifetime agoraphobia (κ = 0.43, *p* = 0.09) or generalized anxiety disorder (κ = 0.57, *p* = 0.06).

#### Structured clinical interview for diagnostic and statistical manual-5th personality disorders

The structured clinical interview for DSM-5 personality disorders (SCID-5-PD) is a semi-structured interview and was used to assess diagnostic symptoms of personality disorders from the DSM-5 by a trained assessor (First et al., 2015b). All traits of personality disorders were coded by the assessor as 0 (does not meet criteria), 1 (subthreshold), or 2 (threshold). The severity of symptoms for each disorder was calculated by summing the ratings of 0, 1, and 2 for all diagnostic criteria for each personality disorder. Categorical diagnoses of personality disorders were rated dichotomously as 0 (below threshold and did not meet criterion) or 1 (above threshold and met full criteria for presence of disorder). Inter-rater reliability was assessed by a blind rater randomly rating 8% of SCID-PD interviews *via* recorded interviews. Inter-rater reliability on total personality disorder symptoms was evaluated using intraclass correlation coefficients (ICCs) with Cohen’s κ analyses. There was agreement among the different raters for the personality disorders (all κ = 1, *p* < 0.001).

#### Demographics

A self-report measure developed for this study was used to obtain demographic and descriptive information, including age, ethnicity, marital status, and income.

#### Misophonia Questionnaire

This is a three-part self-report questionnaire that assesses misophonia symptom presence, resulting emotions and behaviors, and the overall severity of sound sensitivities (Wu et al., 2014). The first part of the scale, the Misophonia Symptom Scale, examines the presence of specific sound sensitivities to different types of sound stimuli (e.g., “people eating,” or “rustling”). For the present study, the mean score for the Misophonia Symptom Scale was 18.4 (*SD* = 6.9). The second part, the Misophonia Emotions and Behaviors Scale, examines emotional and behavioral reactions associated with misophonia. For the present study, the mean score for the Misophonia Emotion and Behaviors Scale was 20.2 (*SD* = 8.0). The first two parts are rated on a scale from 0 (not at all true) to 4 (always true). The third section, named the Misophonia Severity Scale, allows the participant to rate their sound sensitivity on a scale from 1 (minimal) to 15 (very severe). For the present study, the mean score for the symptom severity score was 7.4 (*SD* = 2.6). Finally, the total score for the Misophonia Questionnaire (MQ) was calculated by summing the first two scales, with scores ranging from 0 to 68. Cronbach’s α = *0.88* in this study. The mean total MQ score was 38.6 (*SD* = 13.3) in this study.

#### Medical History Questionnaire

A self-report questionnaire was developed by the study authors to assess participant medical history. The questionnaire assesses a broad array of lifetime medical health problems in participants and family members, including multiple types of developmental problems, neurodevelopmental disorders, neurocognitive disorders, neurological conditions, gastrointestinal problems, sensory processing difficulties, cardiac conditions, kidney conditions, and lung conditions (see Figures 9–17 for details).

### Procedure

Interested individuals were directed to an online screening questionnaire where they provided information about their age, vision, and ability to read in English. They also completed the MQ (Wu et al., 2014). Prospective participants were then screened by telephone using the M.I.N.I. (version 7.0.2; Sheehan et al., 1998) to exclude individuals with a current psychotic disorder, current mania, or current anorexia nervosa. Upon arriving at the laboratory or joining virtually (through Zoom or WebEx), eligible participants provided informed consent, and completed diagnostic interviews and self-report questionnaires with a trained clinical assessor. After completing all study measures, participants were debriefed and received financial compensation for their participation.

### Data analytic plan

#### Outcome variables

All analyses were conducted using SPSS (version 27). For the primary analyses, frequencies were calculated for psychiatric diagnoses and history of medical diagnoses.

#### Missing data and outliers

There were no outliers in these variables, enabling analyses to include all 207 participants. Missing values were not included in analyses.

#### Analytic strategy

Alpha was set *a priori* at a level of 0.05, two-tailed. Point-biserial correlation analyses were conducted to investigate the relationships between misophonia symptom severity (MQ total score; Wu et al., 2014) and (a) categorical psychiatric diagnoses and (b) history of medical health problems. Pearson correlation analyses were also used to investigate the relationships among misophonia symptom severity and measures of psychological functioning, including self-reported severity of psychiatric symptoms and severity of symptoms across personality disorders. To account for the multiple correlation analyses, we report results before and after conducting a Bonferroni correction.

#### Model specification

For secondary analyses, hierarchical linear regression models were conducted to further explore the relationships among misophonia symptom severity and the (a) categories of disorders and (b) specific diagnoses that had significant relationships with misophonia as suggested by univariate analyses. To examine which categories of disorders were the strongest multivariate predictors of misophonia symptom severity (MQ total score computed by summing subscales 1 and 2; Wu et al., 2014), the first model included as predictors dichotomous variables representing whether a participant met full criteria for any current OC related disorders, mood disorders, and anxiety disorders. In the second model, the current DSM-5 disorders that had significant univariate associations were tested as predictors of misophonia severity. In the third model, the symptom severity for avoidant personality disorder, dependent personality disorder, obsessive compulsive personality disorder (OCPD), paranoid personality disorder, schizoid personality disorder, narcissistic personality disorder, and borderline personality disorder (BPD) were tested as predictors. In the fourth exploratory model, the current specific disorders that had significant, direct effects on misophonia from the previous models were tested as predictors. Based on findings in previous studies (e.g., Wu et al., 2014) and significant correlations in the present study, age and sex assigned at birth were entered as planned covariates in Step 1 in all hierarchical regression models, and the other predictors entered in Step 2 with the total score of the MQ entered as the dependent variable.

Before analyses were conducted, tests of assumptions for regressions were conducted. There was linearity as assessed by partial regression plots and a plot of studentized residuals against the predicted values. Independence of residuals was assessed using Durbin-Watson statistics, which ranged from 1.90 to 1.98. Homoscedasticity was assessed by visual inspection of a plot of studentized residuals versus unstandardized predicted values. No evidence of multicollinearity was observed, as assessed by tolerance values greater than 0.1. The assumption of normality was met, as assessed by Q-Q Plot.

## Results

### Descriptive analyses

**FIGURE 1:**
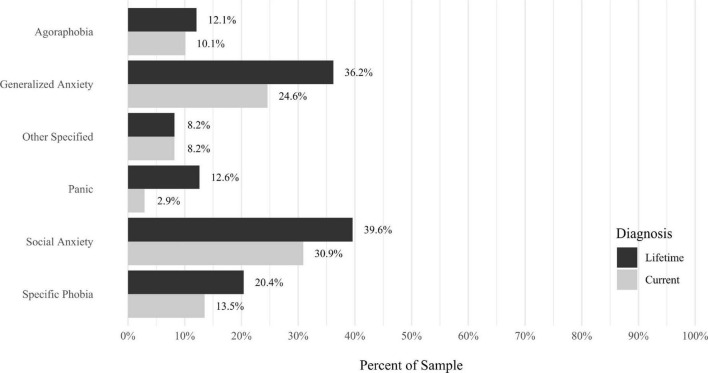
Rates of psychiatric disorders: anxiety disorders.

**FIGURE 2:**
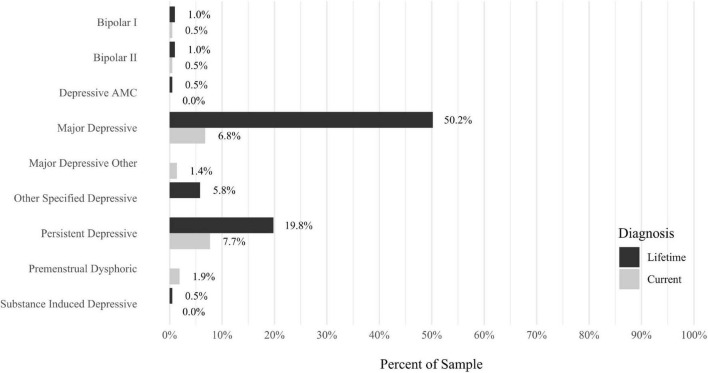
Rates of psychiatric disorders: moods disorders.

**FIGURE 3:**
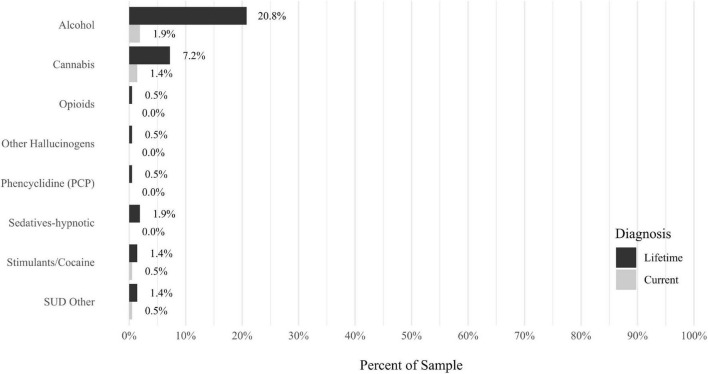
Rates of psychiatric disorders: substance disorders.

**FIGURE 4:**
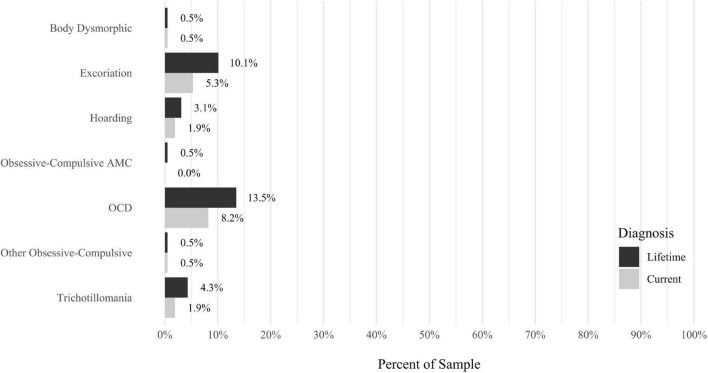
Rates of psychiatric disorders: obsessive compulsive disorders.

**FIGURE 5:**
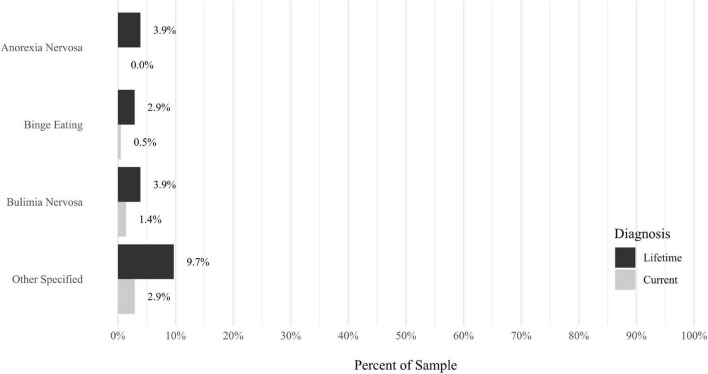
Rates of psychiatric disorders: eating disorders.

**FIGURE 6:**
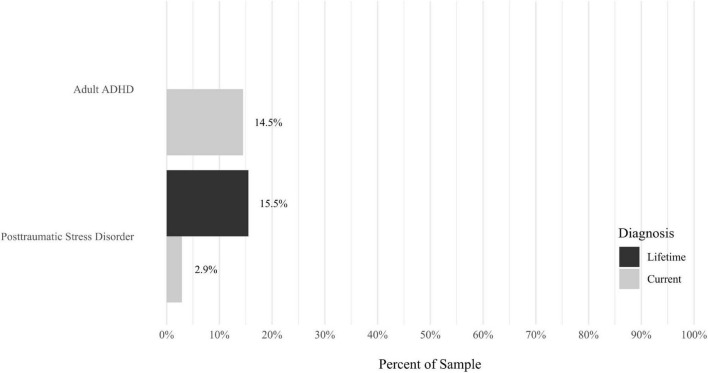
Rates of psychiatric disorders: other disorders.

**FIGURE 7:**
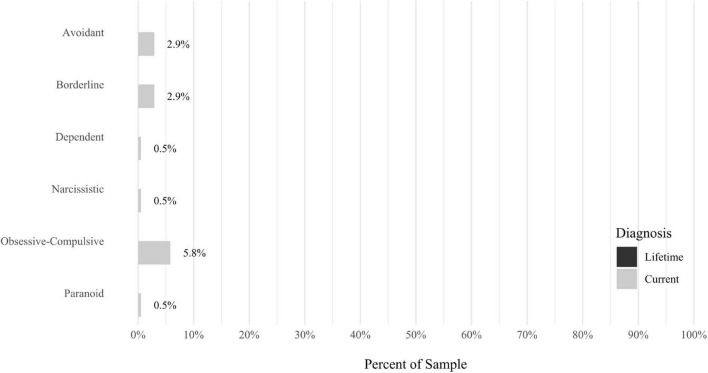
Rates of psychiatric disorders: personality disorders.

**FIGURE 8:**
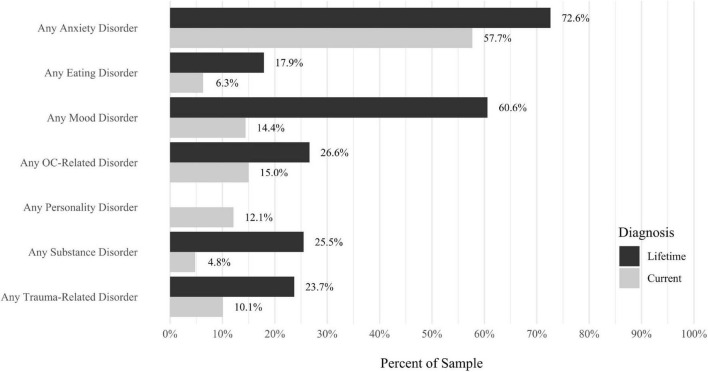
Rates of psychiatric disorders: overall disorders. *N* = 207. All psychiatric diagnoses were determined with the Structured Clinical Interview for DSM-5 (SCID-5; First et al., 2015a). “Current” indicates a current diagnosis and “Lifetime” indicates a diagnosis during a participant’s lifetime. AMC, due to another medical condition. Disorders were not listed if they had a prevalence rate of 0% for both the Lifetime and Current diagnoses. If a disorder is not present in the Current or Lifetime categories, the disorder was not assessed as part of the structured interview.

**FIGURE 9:**
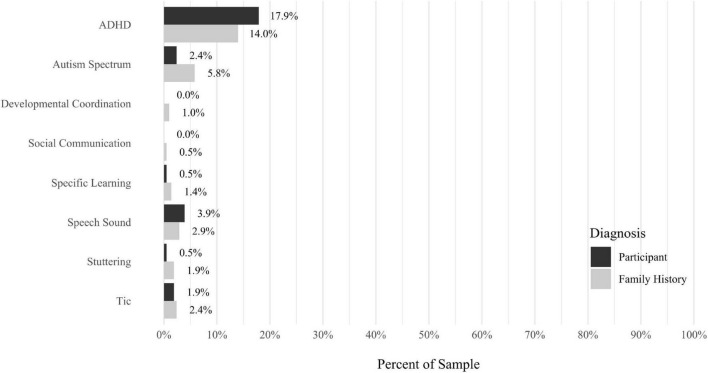
Rates of medical health problems: neurodevelopmental disorders.

**FIGURE 10:**
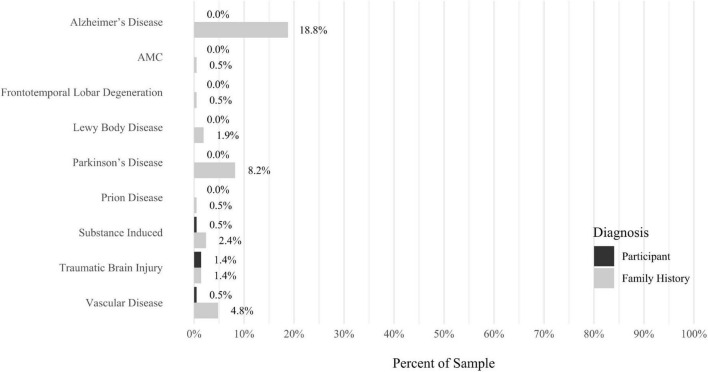
Rates of medical health problems: neurocognitive disorders.

**FIGURE 11:**
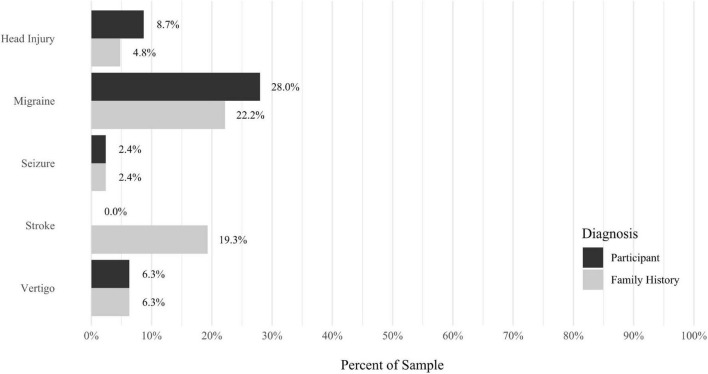
Rates of medical health problems: neurological conditions.

**FIGURE 12:**
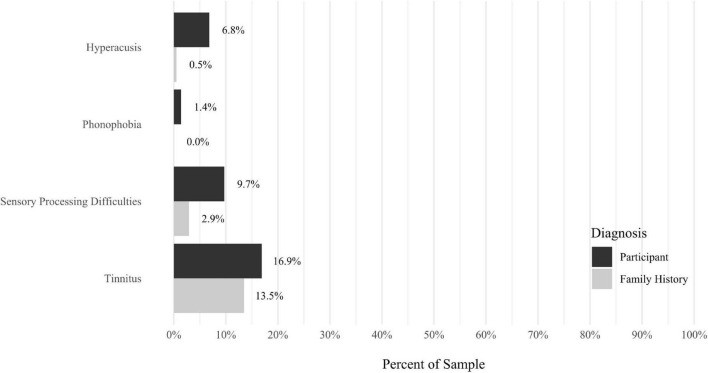
Rates of medical health problems: sensory processing difficulties.

**FIGURE 13:**
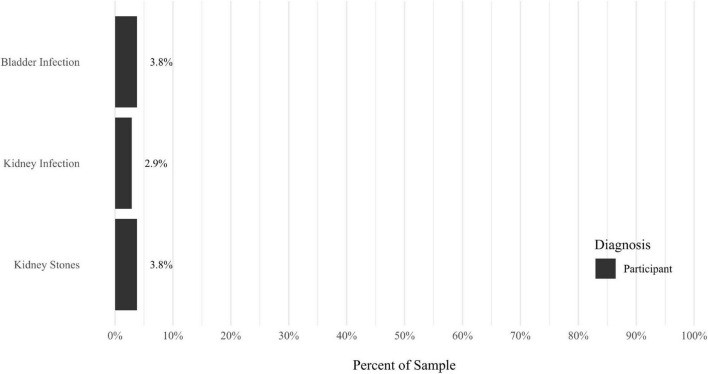
Rates of medical health problems: kidney problems.

**FIGURE 14:**
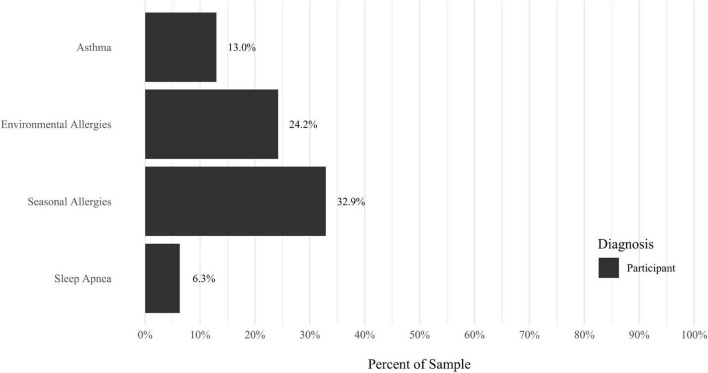
Rates of medical health problems: lung problems.

**FIGURE 15:**
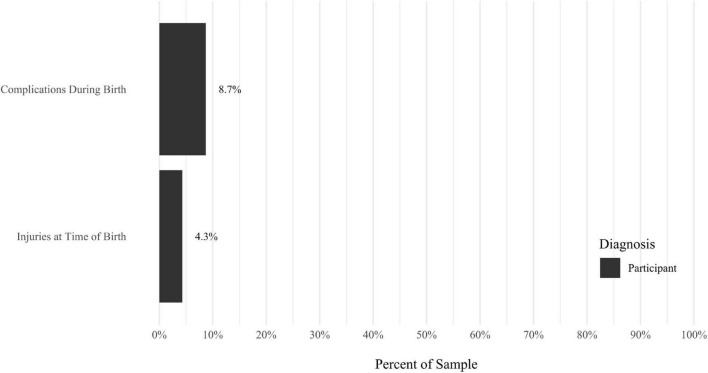
Rates of medical health problems: birth problems.

**FIGURE 16:**
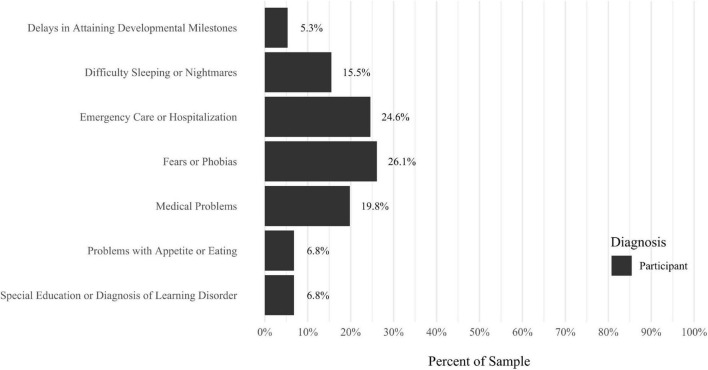
Rates of medical health problems: problems. As a child.

**FIGURE 17:**
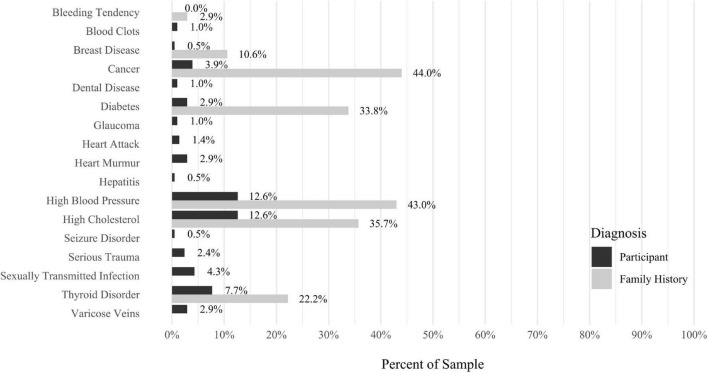
Rates of medical health problems: other problems. *N* = 207. Medical health problems were not listed if they had a prevalence rate of 0% for both the “Participant” and “Family History” categories. AMC, caused by another medical condition.

Anxiety disorders were the most prevalent category of disorder, with 56.9% of the sample meeting the full criteria for at least one current anxiety disorder (*n* = 120). The most commonly diagnosed specific anxiety disorders were social anxiety disorder (30.9%; *n* = 64) and generalized anxiety disorder (24.6%; *n* = 51). Mood disorders were the second most prevalent type of disorder, with 14.2% of the sample meeting full criteria for at least one current mood disorder (*n* = 30). The most commonly diagnosed mood disorders were persistent depressive disorder (7.6%; *n* = 16) and major depressive disorder (6.6%; *n* = 14). Please refer to Figures 1–8 for detailed rates of lifetime and current DSM-5 psychiatric disorders. Detailed rates of medical health history problems are listed in Figures 9–17. The three most commonly reported medical health problems for participants were seasonal allergies (32.2%; *n* = 68), acid reflux (30.8%; *n* = 65), and migraines (27.5%; *n* = 58). The three most common family medical health history items endorsed by participants were cancer (43.1%; *n* = 91), acid reflux (42.8%; *n* = 89), and high cholesterol (35.7%; *n* = 74).

### Correlation analyses

Results from correlational analyses are presented in Tables 2–5. For SCID-5 diagnoses, current OCD, major depressive disorder, persistent depressive disorder, stimulants/cocaine use disorder, panic disorder, agoraphobia, social anxiety disorder, specific phobia, and generalized anxiety disorder all had significant, positive correlations with misophonia symptom severity (*p*s < 0.05). In addition, lifetime history of OCD, major depressive disorder, persistent depressive disorder, alcohol use disorder, hallucinogens use disorder, panic disorder, agoraphobia, social anxiety disorder, and generalized anxiety disorder had significant, positive correlations with misophonia symptom severity (*p*s < 0.05). However, using a Bonferroni correction and corresponding alpha of 0.001, lifetime history of major depressive disorder and persistent depressive disorder was the only disorders significantly correlated with misophonia severity, and the correlation with current OCD was marginally significant (*p* = 0.001).

**TABLE 2:** Correlations between total score on the Misophonia Questionnaire (MQ) and categories of current and lifetime DSM-5 psychiatric diagnoses.

Variable	*r*
1. MQ Total	−
2. Any Current Anxiety	0.27**
3. Any Lifetime Anxiety	0.27**
4. Any Current Mood	0.13
5. Any Lifetime Mood	0.31**
6. Any Current Substance	0.11
7. Any Lifetime Substance	0.12
8. Any Current OC-Related	0.22**
9. Any Lifetime OC-Related	0.23**
10. Any Current Eating	0.05
11. Any Lifetime Eating	0.02
12. Any Current Impulse	0.14*
13. Any Current Trauma-Related	0.06
14. Any Lifetime Trauma-Related	0.18*

*p < 0.05; **p < 0.01.

**TABLE 3:** Pearson’s correlations between total score on the Misophonia Questionnaire (MQ) and specific DSM-5 psychiatric diagnoses.

Variable	MQ total
1. MQ Total	−
2. Bipolar I Lifetime	0.09
3. Bipolar I Current (Past Month)	0.06
4. Bipolar II Lifetime	−0.00
5. Bipolar II Current (Past Month)	−0.00
6. MDD Lifetime	0.26**
7. MDD Current (Past Month)	0.16*
8. PPD Lifetime	0.27**
9. PDD Current (Past 2 Years)	0.19**
10. Alcohol Lifetime	0.14*
11. Alcohol Current (Past 12 Months)	0.08
12. Sedative-Hypnotic Lifetime	−0.00
13. Cannabis Lifetime	−0.02
14. Cannabis Current (Past 12 Months)	−0.01
15. Stimulants/Cocaine Lifetime	0.06
16. Stimulants/Cocaine Current (Past 12 Months)	0.16*
17. Opioids Lifetime	−0.05
18. Panic Lifetime	0.19**
19. Panic Current (Past Month)	0.17*
20. Agoraphobia Lifetime	0.21**
21. Agoraphobia Current (Past 6 Months)	0.19**
22. Social Anxiety Lifetime	0.18**
23. Social Anxiety Current (Past 6 Months)	0.16*
24. Specific Phobia Lifetime	0.13
25. Specific Phobia Current (Past 6 Months)	0.15*
26. Generalized Anxiety Lifetime	0.21**
27. Generalized Anxiety Current (Past 6 Months)	0.17*
28. Obsessive Compulsive Lifetime	0.21**
29. Obsessive Compulsive Current (Past Month)	0.23**
30. Hoarding Lifetime	−0.07
31. Hoarding Current (Past Month)	−0.25
32. Body Dysmorphic Lifetime	0.03
33. Trichotillomania Lifetime	0.07
34. Trichotillomania Current (Past Month)	−0.02
35. Excoriation Lifetime	0.11
36. Excoriation Current (Past Month)	0.14
37. Anorexia Nervosa Lifetime	0.04
38. Bulimia Nervosa Lifetime	0.03
39. Bulimia Nervosa Current (Past 3 Months)	−0.05
40. Binge Eating Lifetime	0.04
41. Binge Eating Current (Past 3 Months)	0.12
42. Avoidant Food Intake Past Month	0.09
43. Intermittent Explosive Current (Past 12 Months)	0.09
44. Adult ADHD	0.14*
45. PTSD Lifetime	0.13
46. PTSD Current (Past Month)	0.13

*p < 0.05; **p < 0.01. Correlations were not included if they could not be computed due to a constant variable (*N* = 1).

**TABLE 4:** Correlations between total score on the Misophonia Questionnaire (MQ) and personality disorder (PD) dimensional profiles.

Variable	MQ total
1. MQ Total	−
2. Avoidant PD	0.19**
3. Dependent PD	0.16*
4. Obsessive Compulsive PD	0.25**
5. Paranoid PD	0.15
6. Schizotypal PD	0.10
7. Schizoid PD	0.21**
8. Histrionic PD	0.04
9. Narcissistic PD	0.14*
10. Borderline PD	0.29**

All personality disorder dimensional profiles were determined by the sum of the scores from the items of each PD (0, 1, or 2) from the structured clinical interview for DSM-5 personality disorders (SCID-5-PD; First et al., 2015b).

*p < 0.05; **p < 0.01.

**TABLE 5:** Correlations between total score on the Misophonia Questionnaire (MQ) and medical health history items.

Variable	MQ total
1. MQ Total	−
2. Language Disorder	−0.08
3. Speech Sound Disorder	−0.05
4. Childhood-Onset Fluency Disorder	0.06
5. Autism Spectrum Disorder	0.01
6. ADHD	0.19**
7. Specific Learning Disorder	−0.08
8. Tic Disorder	0.04
9. Vascular Disease	0.09
10. Traumatic Brain Injury	0.03
11. Substance Induced Neurocognitive Disorder	−0.03
12. Head Injury	0.12
13. Migraine	0.15*
14. Seizure	−0.01
15. Vertigo	0.02
16. Gallbladder Disease	0.12
17. Gastritis/Ulcer Disease	0.02
18. Acid Reflux	0.17*
19. Jaundice	−0.06
20. Hemorrhoids	0.05
21. Sensory Processing Disorder	0.12
22. Tinnitus	0.18**
23. Hyperacusis	0.17*
24. Phonophobia	0.11
25. Breast Disease	0.03
26. Cancer	0.04
27. Diabetes	−0.16*
28. High Cholesterol	0.12
29. Heart Murmur	0.03
30. Heart Attack	−0.12
31. High Blood Pressure	0.03
32. Hepatitis	0.08
33. Glaucoma	0.10
34. Dental Disease	0.10
35. Kidney Infection	0.08
36. Bladder Infection	0.01
37. Kidney Stones	0.02
38. Thyroid Disorder	0.04
39. Varicose Veins	−0.01
40. Seizure Disorder	−0.01
41. Sleep Apnea	0.01
42. Asthma	0.01
43. Seasonal Allergies	0.04
44. Environmental Allergies	−0.07
45. Blood Clots	0.13
46. Serious Trauma	0.14
47. Sexually Transmitted Infection	−0.10

*p < 0.05; **p < 0.01.

For the assessment of medical health problems, a self-reported history of migraines, acid reflux, tinnitus, and hyperacusis all had positive, significant correlations with misophonia symptom severity before Bonferroni correction (*p*s < 0.05). In contrast, a history of diabetes was significantly negatively correlated with misophonia symptom severity (*p*s < 0.05). However, using a Bonferroni correction and corresponding alpha of 0.001, misophonia severity was no longer significantly correlated with any medical health history variable.

### Hierarchical regression analyses

First, we examined which categories of psychiatric disorders were the best predictors of misophonia symptom severity. To accomplish this, we conducted a hierarchical regression with age and sex as covariates in Step 1, and each of the categories of disorders that were significantly positively correlated with misophonia symptom severity in univariate analyses: any current anxiety, OC-related, or mood disorder in Step 2. The model with age and sex predicting misophonia symptom severity in Step 1 was significant [*R*^2^ = 0.06, *F*(2, 204) = 7.00, *p* = 0.001; adjusted *R*^2^ = 0.06]. The full model of age, sex, and meeting full criteria for any current anxiety, OC-related, or mood disorder significantly predicted misophonia symptom severity in Step 2 [*R*^2^ = 0.16, *F*(2, 201) = 7.7, *p* < 0.001; adjusted *R*^2^ = 0.14]. The addition of these categories of current diagnoses over and above age and sex led to a significant increase in *R*^2^ of 0.10, *F*(3, 201) = 7.74, *p* < 0.001. Specifically, results from coefficient analyses revealed that sex (*p* = 0.024), age (*p* = 0.002), meeting full criteria for any current OC-related disorder (*p* = 0.017), and meeting full criteria for any anxiety disorder (*p* = 0.007) each had significant, direct effects on misophonia symptom severity, controlling for the effects of the other variables. Results from coefficient analyses are presented in Table 6.

**TABLE 6:** Coefficient statistics from the full models of the four regression analyses.

	Regression	*B*	*t*	*p*
1	Sex	0.15	2.27	0.02*
	Age (years)	0.20	3.07	0.00**
	Any Current OC-Related	0.16	2.41	0.02*
	Any Current Mood	0.11	1.69	0.09
	Any Current Anxiety	0.19	2.74	0.01*
2	Sex	0.19	2.88	0.00**
	Age (years)	0.21	3.25	0.00**
	Current Obsessive Compulsive Disorder	0.20	2.96	0.00**
	Current Major Depressive Disorder	0.01	0.09	0.93
	Current Persistent Depressive Disorder	0.15	2.25	0.03*
	Current Panic Disorder	0.16	2.56	0.01*
	Current Agoraphobia	0.12	1.89	0.06
	Current Social Anxiety Disorder	0.06	0.86	0.39
	Current Generalized Anxiety Disorder	0.08	1.11	0.27
3	Sex	0.17	2.55	0.01*
	Age (years)	0.18	2.68	0.01*
	Avoidant Dimensional Profile	0.03	0.42	0.67
	Dependent Dimensional Profile	0.02	0.21	0.83
	OCPD Dimensional Profile	0.15	2.18	0.03
	Paranoid PD Dimensional Profile	−0.05	−0.66	0.51
	Schizoid Dimensional Profile	0.08	1.09	0.28
	Narcissistic PD Dimensional Profile	0.09	1.21	0.23
	Borderline PD Dimensional Profile	0.21	2.63	0.01*
4	Sex	0.19	2.91	0.00**
	Age (years)	0.20	3.17	0.00**
	Current Obsessive Compulsive Disorder	0.17	2.64	0.01*
	Current Persistent Depressive Disorder	0.10	1.44	0.15
	Current Panic Disorder	0.15	2.36	0.02*
	OCPD Dimensional Profile	0.10	1.53	0.13
	BPD Dimensional Profile	0.17	2.38	0.02*

All psychiatric diagnoses were determined with the structured clinical interview for DSM-5 (First et al., 2015a). *p < 0.05. Personality disorder dimensional profiles were determined by the sum of the scores from the items of each PD (0, 1, or 2) from the structured clinical interview for DSM-5 personality disorders (SCID-5-PD; First et al., 2015b). Coefficient statistics from the full models (step 2) of the four regressions with the total score of the Misophonia Questionnaire (MQ): 1. Any current disorders composite variables; 2. Specific psychiatric diagnoses; 3. Dimensional profiles of personality disorders; 4. The best predictors from the specific psychiatric diagnoses and personality disorders. **p < 0.01.

Second, we examined which specific psychiatric diagnoses were the strongest predictors of misophonia symptom severity. To accomplish this, we conducted a hierarchical regression with age and sex as covariates in Step 1, and each of the specific disorders that were significantly positively correlated with misophonia symptom severity in univariate analyses: OCD, major depressive disorder, persistent depressive disorder, panic disorder, agoraphobia, social anxiety disorder, and generalized anxiety disorder in Step 2. The first model with age and sex predicting misophonia symptom severity in Step 1 was significant (see above in first regression results). The full model of age, sex, and the current DSM-5 diagnoses significantly predicted misophonia symptom severity in Step 2 [*R*^2^ = 0.22, *F*(9, 197) = 6.08, *p* < 0.001; adjusted *R*^2^ = 0.18]. The addition of these specific current diagnoses over and above age and sex led to a significant increase in *R*^2^ of 0.15, *F*(7, 197) = 5.51, *p* < 0.001. Specifically, results from coefficient analyses revealed that sex (*p* = 0.004), age (*p* = 0.001), OCD (*p* = 0.004), persistent depressive disorder (*p* = 0.025), and panic disorder (*p* = 0.011) each had significant, direct effects on misophonia symptom severity, controlling for the effects of the other variables. Results from coefficient analyses are presented in Table 6.

Third, we examined which personality disorder symptoms were the best predictors of misophonia symptom severity. To accomplish this, we conducted a hierarchical regression with age and sex as covariates in Step 1, and, in Step 2, symptom severity of each of the personality disorders that were significantly positively correlated with misophonia symptom severity in univariate analyses: avoidant, dependent, obsessive compulsive, paranoid, schizoid, narcissistic, and borderline. The first model with age and sex predicting misophonia symptom severity in Step 1 was significant (see above in first regression results). The full model of age, sex, and severity of personality disorder symptoms significantly predicted misophonia symptom severity in Step 2 [*R*^2^ = 0.19, *F*(9,197) = 5.08, *p* < 0.001; adjusted *R*^2^ = 0.15]. The addition of personality disorder symptom severity over and above age and sex led to a significant increase in *R*^2^ of 0.12, *F*(7,197) = 4.30, *p* < 0.001. Specifically, results from coefficient analyses revealed that sex (*p* = 0.011), age (*p* = 0.008), and the severity of OCPD (*p* = 0.030) and BPD (*p* = 0.009) each had significant, direct effects on misophonia symptom severity, controlling for the effects of the other variables. Results from coefficient analyses are presented in Table 6.

Last, we examined which psychiatric disorders were the best overall predictors of misophonia symptom severity. To accomplish this, we conducted a hierarchical regression with age and sex as covariates in Step 1, and, in Step 2, psychiatric disorder variables that emerged as the best predictors of misophonia symptom severity in our second and third hierarchical regressions: OCD, persistent depressive disorder, panic disorder, and severity of OCPD and BPD symptoms. The first model with age and sex predicting misophonia symptom severity in Step 1 was significant (see above in first regression results). The full model of age, sex, and psychiatric disorders in Step 2 significantly predicted misophonia symptom severity [*R*^2^ = 0.23, *F*(7,199) = 8.34, *p* < 0.001; adjusted *R*^2^ = 0.20]. The addition of psychiatric disorders over and above age and sex led to a significant increase in *R*^2^ of 0.23, *F*(5,199) = 8.37, *p* < 0.001. Specifically, results from coefficient analyses in Step 2 revealed that sex (*p* = 0.004), age (*p* = 0.002), OCD (*p* = 0.009), panic disorder (*p* = 0.019), and severity of BPD symptoms (*p* = 0.018) each had significant, direct effects on misophonia symptom severity, controlling for the effects of the other variables. Results from coefficient analyses are presented in Table 6.

## Discussion

The purpose of the present study was to advance an empirical understanding of the phenotypic nature of misophonia in a large sample of adults by (a) using the SCID-5 to comprehensively assess the presence of current and lifetime DSM-5 psychiatric disorders, (b) examining whether there are any medical health history problems associated with misophonia, and (c) determining which specific psychiatric disorders may be the strongest predictors of misophonia severity. This is the first large study to both comprehensively assess DSM-5 current and lifetime psychiatric diagnoses using the SCID-5 (First et al., 2015a) and to explore medical health history using an extensive list of health problems in adults with misophonia.

Results indicated that anxiety disorders were, by a wide margin, the most prevalent type of psychiatric disorders observed in this sample. With 56.9% of the sample meeting full criteria for at least one DSM-5 anxiety disorder, participants in the present study had a far higher rate of anxiety disorders than would be expected in the general population worldwide (estimates range from 4.8 to 10.9% globally; for a recent review, see Stein et al., 2022). Although social anxiety disorder and generalized anxiety disorder were the most prevalent anxiety disorders in this sample, multiple anxiety disorders accounted for the high prevalence, suggesting misophonia is not associated with one specific anxiety disorder. Instead, it may be concluded that adults enrolling in a study about misophonia may be most likely to be diagnosed with any of a number of current anxiety disorders, with the most likely disorders being social anxiety disorder and generalized anxiety disorder. The high prevalence of anxiety disorders notwithstanding, participants also had a range of other co-occurring psychiatric disorders, including mood, OC-related, trauma-related, and personality disorders. For each of these categories of disorders, a pattern emerged wherein multiple specific disorders were present, rather than any one disorder. This is congruent with results from multiple previous studies using diagnostic interviews to assess DSM-IV psychiatric disorders (e.g., Erfanian et al., 2019; Jager et al., 2020; Siepsiak et al., 2022). Additionally, univariate analyses in the present study replicated and extended previous findings indicating that misophonia is not uniquely or specifically associated with any one type of psychiatric disorder. Instead, as has been reviewed elsewhere, misophonia symptoms are significantly positively correlated with a wide range of psychiatric disorders (for reviews, see Brout et al., 2018; Potgieter et al., 2019; Swedo et al., 2022).

Studies are needed to elucidate the relationship between the onset of misophonia during childhood or adolescence and the development of anxiety disorders and other mental health problems in adulthood. One hypothesis is that misophonia is an early life vulnerability factor that temporally precedes and increases anxiety, and that difficulties coping with misophonia and anxiety contribute to the subsequent onset of adult mental health problems. An alternative hypothesis is that early life vulnerabilities to anxiety or other mental health problems contribute to the onset of misophonia. Longitudinal and developmental studies are needed to investigate these hypotheses.

In the absence of prospective data addressing the relationship between misophonia and psychiatric disorders, cross-sectional studies using multivariate analyses may provide helpful information. Indeed, in the present study, a series of regression analyses revealed that (after accounting for age and sex), several psychiatric disorders emerged as multivariate predictors of misophonia symptom severity. Among all psychiatric disorders that were correlated with misophonia severity at the univariate level, BPD symptoms, OCD, and panic disorder each significantly predicted misophonia total score at the multivariate level with significant, independent effects. This result suggests the possibility that items on the MQ assessing misophonia severity (i.e., trigger frequency, common emotions, and behavioral responses when triggered) had direct relationships with these disorders beyond the effects of the other disorders, sex, and age. Because this is the first large study of misophonia to assess DSM-5 psychiatric disorders using structured clinical interviews, it is appropriate to cautiously interpret the findings suggesting these three individual disorders may have particularly strong multivariate associations with misophonia. At the same time, it is important to consider how each of these disorders and their underlying symptoms may have specific mechanisms that are directly related to misophonia.

Although Jager et al. (2020) did not report associations between BPD severity and misophonia, a recent study found that BPD severity, diagnosed using structured clinical interviews, was associated with higher misophonia symptoms (Cassiello-Robbins et al., 2020a). It is possible that there are overlapping features of these disorders, and/or that they share similar underlying mechanisms. For example, the BPD diagnostic criterion of marked anger may be particularly likely to be endorsed among individuals with higher levels of misophonia symptoms. Another hypothesis is that difficulties with affective instability and emotion regulation also jointly characterize BPD and misophonia (Guetta et al., 2022b). Additionally, in light of the low rate of participants above threshold for a diagnosis of BPD in the present study (2.9%), it is also possible that individuals who do meet the full criteria for this diagnosis are particularly likely to have higher misophonia symptoms. However, before firm conclusions can be made about the relationship between BPD and misophonia, additional studies are needed to better understand, at the item level, which BPD symptoms are differentially associated with misophonia.

The relationship between misophonia and OCD also has been previously studied. In Jager et al. (2020), very few participants met the diagnostic criteria for OCD (2.8%). Using the M.I.N.I (Sheehan et al., 1998), Siepsiak et al. (2020a) found that 6.0% of individuals with misophonia met the criteria for OCD, compared to 8.0% in a clinical control group with auditory over-responsivity. In another study using the M.I.N.I. (Sheehan et al., 1998), Erfanian et al. (2019) reported that 11.5% of adults with misophonia (*n* = 6) met the criteria for OCD. Cassiello-Robbins et al. (2020a), using the SCID-I (First et al., 1995), observed that 6.1% of adults met the full criteria for OCD. Among these studies, it is noteworthy that the largest sample (*N* = 575; Jager et al., 2020) had the lowest rate of OCD, in comparison to the other studies, which each had samples below 100 participants and somewhat higher rates. Findings from the present study indicated that 8.2% of participants had a current diagnosis and 13.5% had a lifetime diagnosis of OCD. These studies together do not suggest that OCD is a specific psychiatric disorder expected to co-occur with misophonia but do support the hypothesis that rates of current OCD may be higher in those with misophonia than in general population estimates worldwide (1.1%; Fawcett et al., 2020).

Others have reported significant positive correlations between misophonia symptom severity and OCD symptoms (e.g., Wu et al., 2014). Cusack et al. (2018) found that self-reported OCD symptoms partially mediated the relationship between anxiety sensitivity and misophonia. However, the relationship between OCD and misophonia may be complex, in light of results from McKay et al. (2017), who reported that misophonia symptom severity was positively correlated with some and *negatively* correlated with other features of OCD. Consistent with the notion that some but not all features of OCD may be common in misophonia, researchers have observed that traits of OCPD, but not OCD, are more common in misophonia (Jager et al., 2020).

One influential early study with 42 outpatients found that 52.4% of the sample met the criteria for OCPD, leading the authors to state that misophonia may be considered an OC – related disorder (Schröder et al., 2013). However, much lower rates of OCPD have been observed in more recent studies using a small community sample (10.2%; Cassiello-Robbins et al., 2021), a large sample of treatment-seeking adult outpatients with misophonia (2.4%; Jager et al., 2020), and in the present study (5.8%). In light of these mixed results, and given the estimated lifetime prevalence of OCPD in large epidemiologic samples (7.8%; Grant et al., 2012), it is important that additional studies are conducted to more clearly understand the relationship between misophonia and OCPD. It is possible that some (but not all) OCPD criterion behaviors are differentially associated with greater misophonia, including, for example, (1) preoccupation with details, rules, lists, order, organization, or schedules, (2) perfectionism that interferes with task completion, (3) over-conscientiousness, (4) inflexibility about matters of morality, ethics, or values, and (5) reluctance to delegate tasks or to work with others. This hypothesis is indirectly supported by Jager et al. (2020), who reported that 23.8% of their sample had OCPD traits, even though only 2.4% met the full criteria for the disorder.

Unlike OCD and OCPD, panic disorder has been studied relatively less in misophonia. It may be that the tendency to be highly distressed by interoceptive sympathetic nervous system cues (e.g., increased heart rate) and to avoid or escape from stimuli that elicit intense anxious arousal are shared features of panic disorder and misophonia. Although speculative, it is also possible that individual differences in transdiagnostic traits such as harm avoidance (McKay et al., 2017) or distress intolerance underlie both misophonia and panic disorder. Alternatively, it may be that the small number of participants with panic disorder in the present study had very high misophonia severity due to chance or an unobserved variable. Conservatively, results pointing to panic disorder as among the strongest predictors of misophonia severity need to be replicated in samples with higher frequencies before clear conclusions can be made.

In addition to investigating psychiatric disorders, this is the first larger scale study to report data assessing lifetime medical health problems among individuals with misophonia. An extensive list of medical problems was used, including developmental, neurological, audiological, cardiac, and other health problems. Before using an alpha correction procedure, results indicated that misophonia symptom severity was modestly but significantly associated with a lifetime history of migraines, acid reflux, tinnitus, and hyperacusis. However, when more conservatively accounting for multiple tests using a Bonferroni correction, no medical health problems were significantly associated with misophonia severity. This conclusion aligns with Jager et al. (2020), who also found no clear pattern of medical health problems associated with misophonia. Despite the findings from these studies, before definitive conclusions are made about medical health problems and misophonia, additional research using more rigorous methodologies is needed (e.g., population level data from electronic medical records, structured health history interviews).

There are a number of limitations in the present study that preclude definitive conclusions. A larger sample would enable a deeper understanding of the possible relationship between misophonia and psychiatric disorders and medical problems that have low base rates. Results from this study do not causally account for the nature of the relationship between any psychiatric disorders and misophonia. It is possible that there are transdiagnostic underlying processes across misophonia and the psychiatric disorders found in the regression analyses to each have direct effects. Difficulties with emotion regulation (i.e., anger regulation; Guetta et al., 2022b) or individual differences in harm avoidance McKay et al. (2017), for example, are two plausible candidate processes that can be examined in future studies. However, until the present study is replicated and prospective studies are conducted in large samples, it is only possible to speculate on such putative underlying mechanisms.

Despite this being one of the largest published studies to date using structured interviews, the study sample size was not large enough to observe high frequencies of certain diagnoses. This may have contributed to low rates of co-occurring disorders and limited statistical power to detect significant effects in our regressions. For example, only six participants met the full criteria for current panic disorder, and six met the full criteria for BPD. Although almost all previous studies examining psychiatric disorders in misophonia have smaller samples than the present study, it will be important to conduct larger future studies to rule out the possibility that findings using multiple regressions from the present study were related, in part, to the small samples of individuals with certain disorders.

Although efforts in the present study were made to diversify participant enrollment, the relative lack of racial, ethnic, gender, and socioeconomic diversity is a limitation that has been present in most studies of misophonia. Indeed, before conclusions can be made about the nature and features of misophonia, it is imperative that researchers recruit diverse samples of individuals that represent all people with misophonia, rather than samples that are primarily White, female, heterosexual, cisgender, and from high-income families.

One approach that can be used in future studies to increase the diversity of study enrollment is the use of population sampling methods that recruit randomly across households nationally. Although such studies require significant resources to complete, this will be a necessary step as science advances to characterize misophonia using increasingly rigorous methods. Such a sampling approach also would help ensure that findings from any given study are not confounded in any way by the geographical location, relative expertise, or any other factor associated with the investigative team and site. Indeed, in the present study, prospective participants contacted the study team directly through online screening found on the laboratory website. Although expedient, there may be participant factors associated with the capability and willingness to locate and enroll in research studies on misophonia. Until random sampling procedures are used, findings from the present study and all previous studies of misophonia should be interpreted with appropriate and reasonable inferences about the generalizability of study findings.

Another limitation of the study is the absence of assessment of several psychiatric disorders not included in the SCID-5. Although the SCID-5 is widely considered a gold standard measure used in large-scale epidemiologic studies, disorders of childhood and autism spectrum disorder were not assessed. To extend findings from the present study, it will be important for researchers to include assessment measures for these disorders in future studies designed to characterize misophonia. A related limitation is the absence of data in the present study with children and adolescents. To better understand the nature and features of misophonia it will be critical for future studies to include samples of children and adolescents, and to assess the onset of misophonia over time longitudinally and in the context of multiple developmental, environmental, and biological factors.

To summarize, the present study is the first to examine the relationship between misophonia and DSM-5 psychiatric disorders comprehensively using the SCID-5. Results indicated that anxiety disorders were the most common kinds of mental health problems associated with misophonia. Replicating and extending previous studies, misophonia symptoms were positively correlated with a wide range of psychiatric disorders, rather than being specifically related to any specific disorder (e.g., McKay et al., 2017; Rouw and Erfanian, 2018; Erfanian et al., 2019; Cassiello-Robbins et al., 2020b; Jager et al., 2020; Guetta et al., 2022b). However, regression analyses revealed that certain disorders were more strongly predictive of misophonia severity, over and above age (older), and sex (female). In addition, this is the largest study to examine the frequency of medical health problems among adults with misophonia. No discernable pattern of medical health history correlates was observed when controlling for multiple comparisons statistically. Results advance an understanding of the nature and features of misophonia in adults.

## Data availability statement

The raw data supporting the conclusions of this article will be made available by the authors, without undue reservation.

## Ethics statement

The studies involving human participants were reviewed and approved by Duke Health Institutional Review Board. The participants provided signed informed consent to participate in this study.

## Author contributions

MR designed and led study implementation, directed analyses, and led manuscript writing. KM led data analysis and co-led manuscript writing. AG assisted with analyses and manuscript preparation. CC-R co-led study implementation and assisted with manuscript editing. RG and JT assisted with study implementation. DA co-led study design and implementation. EF-A assisted with manuscript preparation. LK assisted with study design and implementation. All authors contributed to the article and approved the submitted version.

## Footnotes

1
www.misophonia.duke.edu
